# ROS-1 Positive Pleural Adenocarcinoma Diagnosed by Semirigid Thoracoscope Using Cryo-Probe in a Middle-Aged Lady: A Case Report

**DOI:** 10.7759/cureus.102967

**Published:** 2026-02-04

**Authors:** Jyoti Bajpai, Shubhajeet Roy, Surya Kant

**Affiliations:** 1 Respiratory Medicine, King George’s Medical University, Lucknow, IND; 2 General Surgery, All India Institute of Medical Sciences, Raebareli, Raebareli, IND; 3 Respiratory Medicine, King George's Medical University, Lucknow, IND

**Keywords:** cryo-probe, non-small cell lung cancer, pleural adenocarcinoma, ros-1, thoracoscope

## Abstract

ROS-1 positive non-small cell lung cancer (NSCLC) constitutes a lesser-known yet targetable subcategory of lung malignancies, contributing 1-2% of cases. NSCLC management has been revolutionized by molecular profiling, which employs the identification of driver mutations that permit personalized therapy.

A 40-year-old lady presented with gradual right-sided chest pain, dry cough, breathlessness, weight loss, and loss of appetite. Radiology (CECT) showed a right-sided pleural effusion, and pleural biopsy revealed adenocarcinoma. Pleural cryobiopsy was done by a flexible cryo-probe, passed via the working port of a semirigid thoracoscope till the parietal pleura, followed by its activation to freeze the tissue for 3-5s, to ultimately perform an en-bloc resection. Immunohistochemistry revealed ROS1 positivity, but EGFR and ALK were negative. She was initiated on crizotinib, which led to a remarkable clinical response and tumor regression.

Absolute diagnosis of pleural neoplasia can be daunting because of nonspecific imaging and cytology findings, more so if the pathology is in the fibrotic pleura. Pleural cryobiopsy, which employs a semirigid thoracoscope, makes room for larger and better-preserved tissue samples, enhancing the diagnostic yield in complicated scenarios like ours.

## Introduction

Even until some 15-20 years back, non-small cell lung cancer (NSCLC) was thought to be a singular entity, and the systemic therapy for metastatic NSCLC was quite restricted to platinum-based chemotherapy doublets [1]. The response rates ranged mostly around 20%, and a median survival of only eight months was reported across studies, which showcased the restricted efficacy of such classical cytotoxic regimes [2,3]. With increasing recognition of histopathological subtypes even under the broad heading of NSCLC, the importance of accurate tissue diagnosis has become central to dictating systemic treatment algorithms, especially in the case of platinum-based chemotherapy [1,2,4]. This categorisation has paved the way for further tailored therapeutic regimes, enhancing treatment prognosis and widening the spectrum of therapeutic alternatives.

In cases where NSCLC presents with pleural-predominant disease, reaching a particular diagnosis can be complicated due to nonspecific imaging findings and the poor diagnostic yield of pleural fluid cytology. Pleural biopsy becomes even more important in such scenarios, particularly when there are fibrotic changes in the pleura or when fluid cytology is non-yielding. Cryobiopsy with semirigid thoracoscopy offers larger and better-preserved tissue samples compared to classical forceps biopsy, thereby enhancing diagnostic accuracy while decreasing procedure-associated trauma [1,2,5].

## Case presentation

A lady of age 40 years presented to the Respiratory Medicine outpatient department with right-sided chest pain, which was insidious in onset, progressive, dull aching, intermittent, and without postural or diurnal variation, for the last 2.5 years; dry non-productive cough, with no postural or diurnal variation, for two years; and breathlessness upon mild exertion (mMRC grade II-III) for one year. She also complained of a decrease in appetite and significant unintentional loss of weight for the same duration. There were no complaints of fever, night sweats, or hemoptysis. She did not have a previous history of tuberculosis, she was a non-smoker, and had no history of any significant occupational exposure, or previous history of any malignancy. On examination, the patient was well oriented to time, place, and person, and was stable hemodynamically. Examination of the respiratory system revealed decreased right chest movements. Upon percussion, a stony dull note was heard over the right chest in the middle and inferior zones. Breath sounds and vocal fremitus were severely decreased over the same areas. The rest of the systemic examinations had findings within the normal limits. There was no peripheral lymphadenopathy felt upon palpation.

Based on clinical history and examination, infectious, obstructive, and inflammatory lung diseases were less probable because of the absence of fever, productive cough, wheeze, smoking history, or occupational exposure. The chronic progressive complaints with unilateral pleural presentation and weight loss pointed more towards a malignant pathology.

**Figure 1:**
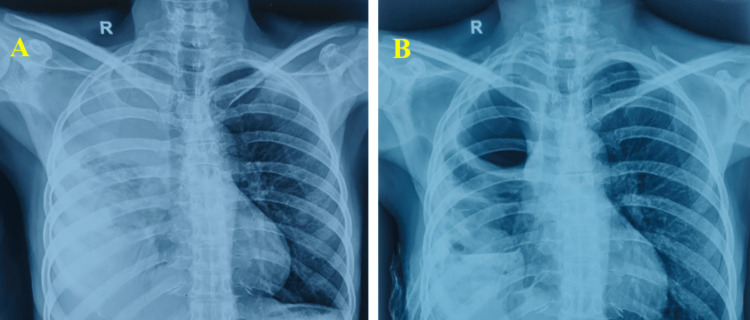
Chest X-ray postero-anterior view: (A) Post bronchoscopy and post pleural tap. (B) Before the first cycle of chemotherapy The chest radiograph demonstrates a large right-sided pleural effusion, seen as a homogeneous opacity occupying the lower and mid zones of the right hemithorax with an obscured right costophrenic angle and a meniscus sign. There is compression of the underlying lung parenchyma, without obvious contralateral mediastinal shift. The appearance is consistent with a significant pleural effusion, correlating with the patient’s pleural-predominant disease.

Initial blood investigations revealed findings all within normal ranges. The chest radiograph showed a right-sided homogenous opacity corresponding to a right-sided pleural effusion (Figure 1).

**Figure 2:**
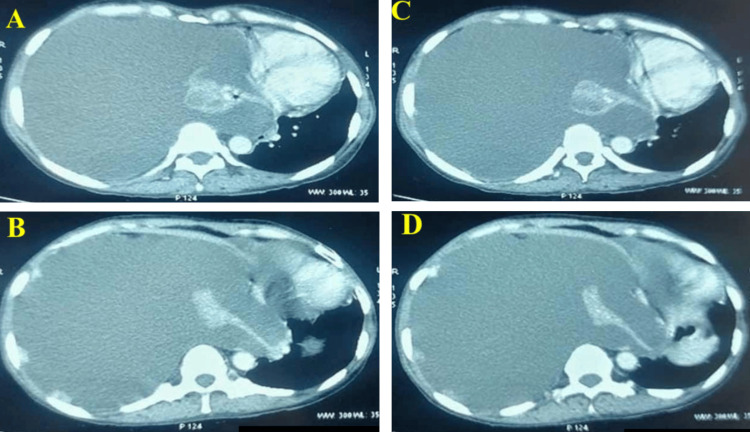
CECT of the thorax (axial view) showing massive right-sided pleural effusion, associated with diffuse pleural thickening and nodularity. (A–D) Axial CECT images of the thorax demonstrate a massive right-sided pleural effusion causing near-complete compression of the underlying lung parenchyma. There is diffuse circumferential pleural thickening involving the parietal pleura, with irregular nodular pleural deposits seen along the costal and diaphragmatic pleura. The pleural thickening appears heterogeneous and enhancing, suggestive of pleural involvement by an underlying pathological process. No discrete endobronchial lesion or focal lung parenchymal mass is identified on these sections.

Contrast-enhanced computed tomography (CECT) of the thorax showed massive right-sided pleural effusion, associated with diffuse pleural thickening and nodularity. No endobronchial or lung parenchymal lesion could be appreciated (Figure 2).

**Figure 3:**
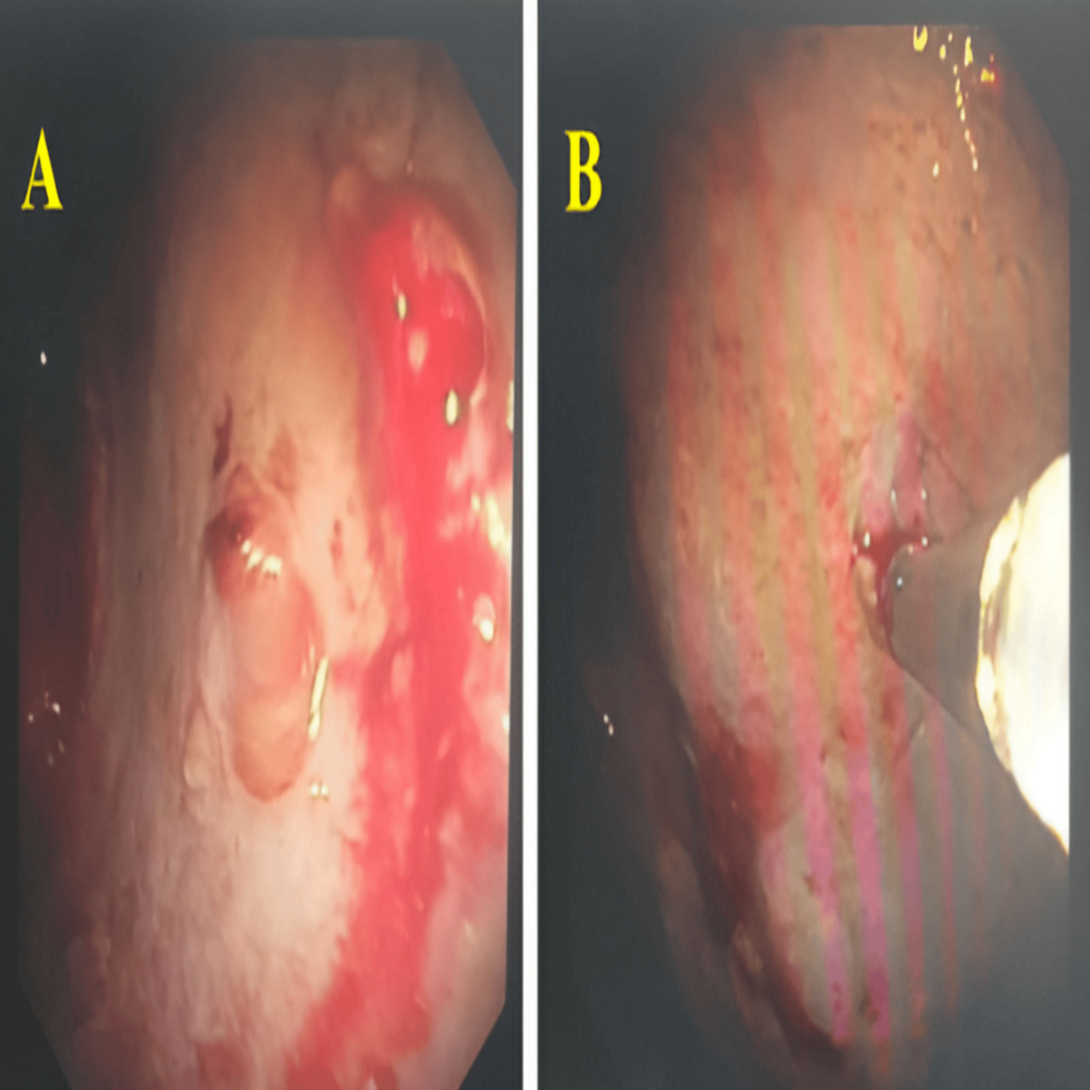
Thoracoscopy showing thickened and fibrosed parietal pleura and multiple-sized nodules (A) Thoracoscopic view of the parietal pleura showing diffuse pleural thickening with a fibrotic, irregular surface, along with multiple nodular lesions of varying sizes distributed over the pleural surface. The pleura appears hyperemic with loss of the normal smooth glistening appearance. (B) Closer thoracoscopic view highlighting the nodular pleural deposits and thickened parietal pleura, with areas of surface irregularity and focal hemorrhagic spots. These findings are suggestive of malignant pleural involvement and guided targeted pleural biopsies.

A diagnostic pleural tap revealed an exudative fluid on Light’s criteria. It was straw-colored, with predominant cells being lymphocytes. Protein and lactate dehydrogenase levels were elevated, and glucose and pH were normal. Adenosine deaminase (ADA) levels were not suggestive of tubercular etiology. Gram stain and bacterial cultures were negative, and acid-fast bacilli smear and GeneXpert for Mycobacterium tuberculosis were non-contributory. Pleural fluid cytological examination did not reveal malignant cells, necessitating further evaluation with thoracoscopic pleural biopsy. Flexible bronchoscopy was done to check for endobronchial lesions, but the findings were non-significant. The patient was taken up for medical thoracoscopy for a persistent undiagnosed right-sided exudative pleural effusion, wherein under local anesthesia and conscious sedation, a single port semi-rigid thoracoscope was introduced on the right hemithorax in the 5th intercostal space along the mid-axillary line till the parietal pleura, which revealed thickened and fibrosed parietal pleura and multiple-sized nodules. These features were indicative of malignancy. Approximately moderate volume pleural fluid was drained to facilitate adequate visualization of the pleural cavity. Multiple pleural biopsies (en bloc) were obtained from representative abnormal areas using a flexible cryoprobe, with a freeze time of 3-5 seconds per biopsy. The sample yield was quite large with minimal bleeding. The procedure was well tolerated. Known complications of semirigid thoracoscopy are bleeding, air leak, infection, pain, hypoxia, and injury to intrathoracic structures; however, no procedure-related complications occurred in our patient (Figure 3).

According to the AJCC 9th edition TNM staging system, the presence of malignant pleural involvement classifies the disease as stage IVA (M1a), irrespective of the primary tumor size or nodal status.

**Figure 4:**
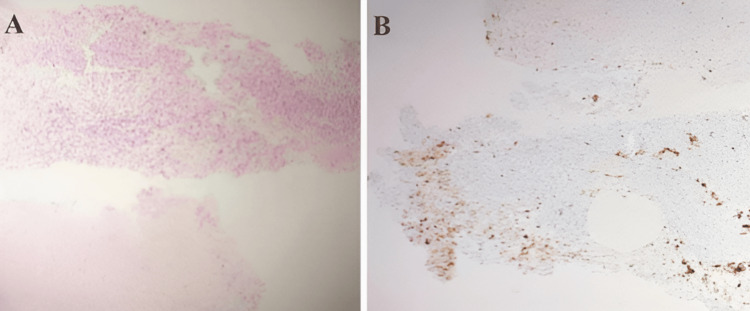
(A) Histopathological examination: Haematoxylin-eosin (H&E) slide suggestive of adenocarcinoma; (B) Immunohistochemistry suggestive of ROS-1 positivity.

The histopathology report suggested non-small cell lung carcinoma-adenocarcinoma subtype, and the immunohistochemistry came back as ROS-1 positive, EGFR-negative, and ALK-negative (Figure 4).

Hence, a diagnosis of ROS-1-positive pleural adenocarcinoma was reached at. The patient was started on crizotinib therapy after ascertaining a good Eastern Cooperative Oncology Group score. It has been six months since initiation of treatment, and she has been put under monthly clinical and 2 monthly radiological follow-up. Upon periodic follow-up, a remarkable clinical prognosis was achieved, marked by a reduction in the symptoms, and improved appetite and functional status.

## Discussion

Lung cancer happens to be one of the most prevalent causes of cancer-related mortalities globally, with somewhat greater than 1.8 million deaths in 2020 attributed to lung cancers [6]. In patients with NSCLC, around 25% possess oncogenic variations, which can be targeted by medications. An example of that is the proto-oncogene tyrosine-protein kinase-1 (ROS-1; c-Ros oncogene-1) gene fusion, which, if identified, makes patients perfect candidates for targeted management [7,8].

Molecular diagnosis and subsequent therapeutic management of lung cancer have undergone a major shift by identification of driver mutations and oncogenic rearrangements. A remarkable step in this aspect is the identification of sensitizing mutations of the epidermal growth factor receptor gene (EGFR), which render them susceptible to EGFR tyrosine kinase inhibitors (EGFR-TKIs) like erlotinib, gefitinib, and afatinib. Such targeted management has showcased greater effectivity contrasted to classical chemotherapy in certain select group of patients. Moreover, the discovery of anaplastic lymphoma kinase (ALK) gene rearrangements, like EML4-ALK, has paved the way for the generation of ALK inhibitors, like crizotinib and ceritinib, which, in a way, have led to the growth of therapeutic alternatives for patients with advanced NSCLC [9,10].

Exact diagnosis of pleural neoplasia may be quite complicated due to manifold factors. Cytological examination of pleural fluid is low in terms of sensitivity, more so in mesothelial hyperplasia, fibrosis, or paucicellular samples [11,12]. Classical forceps biopsies come with an array of limitations in terms of diagnostic yield, more so if there is a presence of thickened or fibrosed pleura, because in such cases, sample tissue acquisition is insufficient or comes with other technical difficulties. Moreover, some anatomical locations, or rather “blind spots,” become quite hard to reach with rigid instruments, and the small size of such samples results in inconclusive results [12].

In the present case, the presence of thickened parietal pleura along with nodularity seen through thoracoscopy led us to choose cryobiopsy over classical forceps biopsy [1]. Cryobiopsy has got a significant upper hand in these cases. It paves the way for larger and better-preserved tissue samples, and that is crucial for reaching at a histological diagnosis and performing advanced molecular diagnostics. Contrasted to classical biopsy with forceps, cryoprobes lead to significantly decreased crush artifact and bleeding, preserving tissue architecture, which is absolutely important for exact histologic subtyping and biomarker analysis. Additionally, cryobiopsy reduces procedure duration and enhances diagnostic yield in cases with suspected pleural neoplasia, especially when traditional methods have considerably higher failure percentages [2,13]. Researches have also exemplified that cryobiopsy samples are ideal not just for routine H&E staining, but also for IHC examinations of slides like TTF-1, napsin A, p40, and cytokeratin spectra, and also for recent advances like the next-generation sequencing (NGS), if and when deemed necessary. This paves the path for personalized oncology diagnosis and therapy [14,15].

Trials have proved the effectivity of crizotinib, a tyrosine kinase inhibitor (TKI) against ROS-1, ALK, and mesenchymal-to-epithelial transition (MET) protein. Hence, it has emerged to be the preferred first-line treatment for ROS-1-positive NSCLC [16]. Although crizotinib persists to be the recommended first-line management, newer drugs with increased blood-brain barrier penetration, like entrectinib and lorlatinib, have shown quite positive results in preventing metastases to the brain. Furthermore, repotrectinib and taletrectinib have shown good results in our fight against resistance, especially the ROS-1-G2032R-fusion variant [17,18]. Studies report that foretinib and repotrectinib are quite effective in G2032R-mutated ROS-1 [19].

## Conclusions

Continuous research into the molecular mechanisms behind the resistance pathways will ensure the advent of more effective newer-generation TKIs with improved potency and longevity. These trials should also strive to experiment combination strategies and personalized therapeutic options to maximize treatment advantages. By improving our knowledge of pathologic prognosis, progression and resistance, we can gradually strive towards more effective, tailored procedures that enhance patient outcomes.
